# Reply to: Burosumab for Tumor‐Induced Osteomalacia: not Enough of a Good Thing

**DOI:** 10.1002/jbmr.4317

**Published:** 2021-05-24

**Authors:** Suzanne M. Jan de Beur, Paul D. Miller, Thomas J. Weber, Munro Peacock, Karl Insogna, Rajiv Kumar, Frank Rauch, Diana Luca, Tricia Cimms, Mary Scott Roberts, Javier San Martin, Thomas O. Carpenter

**Affiliations:** ^1^ Johns Hopkins University School of Medicine Baltimore MD USA; ^2^ Colorado Center for Bone Research Lakewood CO USA; ^3^ Duke University Durham NC USA; ^4^ Indiana University School of Medicine Indianapolis IN USA; ^5^ Yale University School of Medicine New Haven CT USA; ^6^ Mayo Clinic College of Medicine Rochester MN USA; ^7^ McGill University Montreal Canada; ^8^ Ultragenyx Pharmaceutical Inc. Novato CA USA

**To the Editor:**

We appreciate that Dr Hartley and Dr Collins recognize the importance of burosumab as a treatment for patients with tumor‐induced osteomalacia (TIO) and X‐linked hypophosphatemia (XLH). Burosumab is the only US Food and Drug Administration (FDA)‐approved therapy for patients with TIO who have unresectable or unlocalizable tumors. Up until the approval of burosumab, treatment with active vitamin D and phosphate was the standard of care for medical treatment of TIO. However, treatment with these agents must be carefully monitored and titrated to avoid side effects such as secondary hyperparathyroidism, hypercalciuria, and nephrocalcinosis. For these reasons, clinicians aim for blood phosphate levels that are just within the lower limit of normal. The registrational TIO study^(^ ^1^ ^)^ was designed in the context of this traditional approach to treatment and was implemented prior to the availability of robust, long‐term safety data of burosumab in adult patients with XLH.^(^ ^2^, ^3^, ^4^ ^)^ As such, burosumab was initially given at a conservative dose (0.3 mg/kg every 4 weeks) and then titrated to a maximum dose of 2.0 mg/kg every 4 weeks to achieve a serum phosphate level just above the lower limit of normal. Some patients required higher doses to reach the lower limit of normal, and these doses were well tolerated. Based on these data, the FDA has approved burosumab for patients with TIO with doses ranging from 0.5 mg/kg every 4 weeks up to 2.0 mg/kg every 2 weeks.

Although XLH and TIO have the commonality of fibroblast growth factor 23 (FGF23)‐mediated hypophosphatemia, they are different diseases with different underlying etiologies and clinical manifestations. TIO symptoms and physical signs can be more debilitating. As such, it is difficult to directly compare burosumab efficacy across disease states and across clinical studies with different assessments and endpoints. Compared to patients enrolled in the adult XLH Phase 3 clinical trials,^(^ ^2^, ^3^ ^)^ TIO patients had lower serum phosphate, higher FGF23, and more variable histomorphometric parameters of osteomalacia upon study entry. The latter may be explained by differences in study enrollment criteria in which TIO patients continued to receive phosphate/active vitamin D until 2 weeks prior to study entry, whereas XLH patients in the bone biopsy subset were ineligible to participate if they received phosphate/active vitamin D within 2 years prior to study entry.^(^ ^3^ ^)^

Patients with TIO treated with burosumab experienced significant improvements across multiple measures, including some measures of osteomalacia, fracture healing, and patient‐reported pain, fatigue, and physical functioning. Of note, significant changes were seen in the setting of a small study population of 14 patients, and despite the fact that most patients did not reach an effective burosumab dose until the end of the 16‐week titration period. Because of the titration period, patients were on an effective burosumab dose for only 32 weeks at the time of the second bone biopsy, which may explain why significant improvements in some, but not all, osteomalacia parameters were observed. With longer burosumab treatment, we would expect to see greater improvements in these measures as well. Similarly, 33% of fractures were fully healed after 144 weeks of treatment. In this study, fractures were assessed by full‐body bone scan, whereas the XLH studies^(^ ^2^, ^3^ ^)^ used radiographic skeletal survey at baseline with targeted radiographs to assess fracture healing thereafter. Given the differences in sensitivity of these techniques, it simply takes longer for fractures to appear fully healed on bone scans than with x‐rays.^(^ ^5^ ^)^

Burosumab has been shown to be well tolerated and effective inside the limits of a clinical trial. Now that burosumab is approved for patients with TIO, we are excited to see the impact this treatment option has for patients in the rea‐world and over longer periods of time. Ultragenyx (Novato, CA, USA) has initiated disease monitoring programs for patients with TIO (https://clinicaltrials.gov/ct2/show/NCT04783428) and XLH (https://clinicaltrials.gov/ct2/show/NCT03651505) to gather data on the natural history of these diseases and the burden of illness in treated and untreated patients as well as the long‐term safety and efficacy of burosumab. We hope that real‐world data will continue to inform treatment recommendations so that those that suffer with TIO may have the best possible outcomes.
